# Supplementary figures and images for: Potential for Duplexed, In-Tandem gRNA-Mediated Suppression of Two Essential Genes of Tomato Leaf Curl New Delhi Virus in Crop Plants
Source: Pathogens. 2025 Jul 10;14(7):679. doi: 10.3390/pathogens14070679 (PMC12298162; doi:10.3390/pathogens14070679)

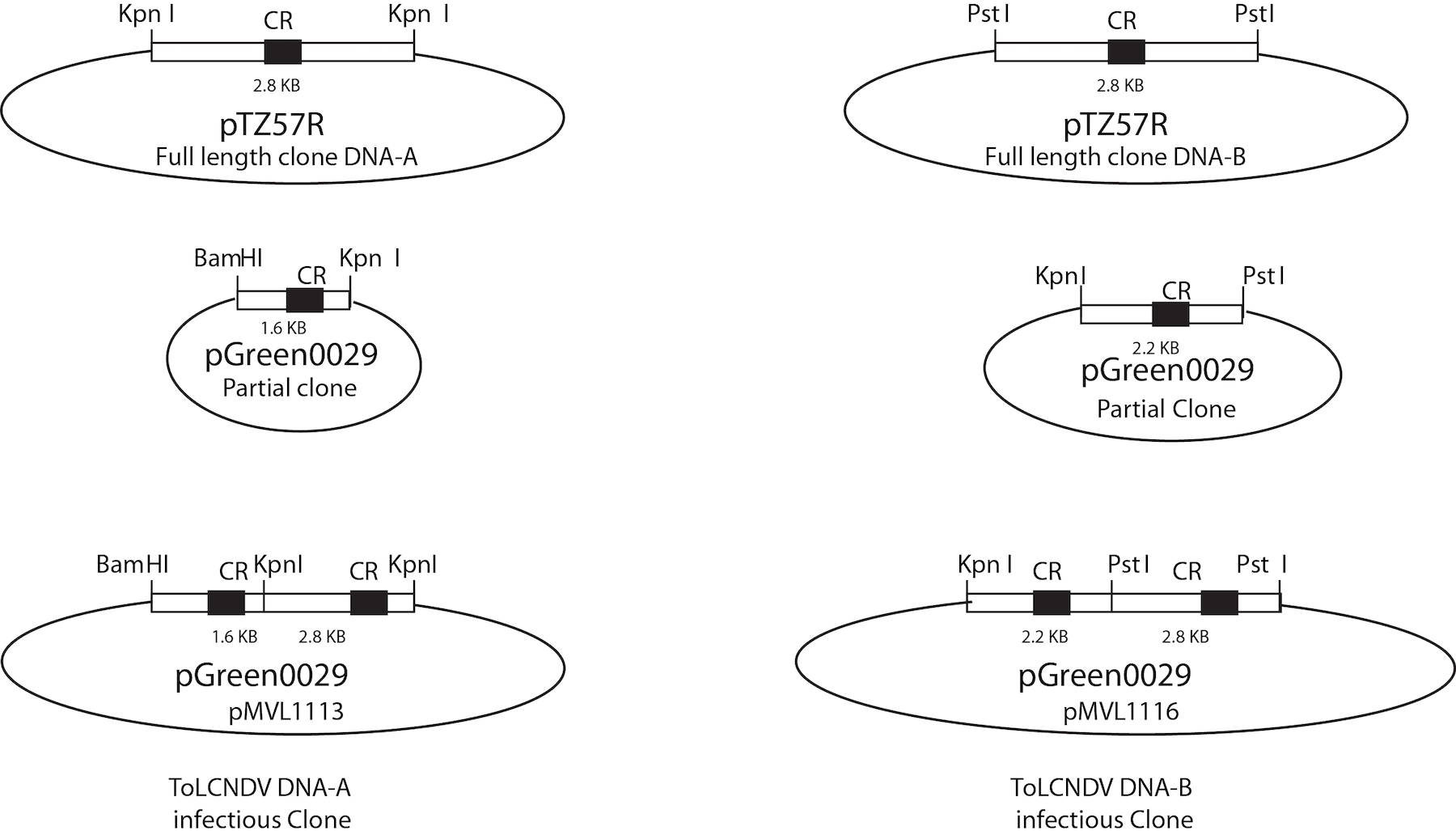

Supplement: Supplementary file 1 [file pathogens-14-00679-s001.zip › pathogens-3618910-supplementary.png]
